# Surgical skills assessment on a vaginal Natural Orifice Transluminal Endoscopic Surgery (vNOTES) simulation box compared to a conventional endoscopic simulation box; the SAVE trial

**DOI:** 10.1007/s00464-025-12389-7

**Published:** 2026-03-02

**Authors:** Rebecca Henschen, Ilse P. W. Bekkers, Jan Baekelandt, Jacques W. M. Maas, Huib A. A. M. van Vliet, Martine M. L. H. Wassen

**Affiliations:** 1 Department of Obstetrics and Gynecology, Zuyderland Medical Center, Henri Dunantstraat 5, 6419 PC Heerlen, The Netherlands; 2 GROW—Research Institute for Oncology and Reproduction, Maastricht University Medical Center+, Universiteitssingel 40, 6229 ER Maastricht, The Netherlands; 3 Department of Obstetrics and Gynecology, Maastricht University Medical Center+, P. Debyelaan 25, 6202 AZ Maastricht, the Netherlands; 4 Department of Obstetrics and Gynecology, Imelda Hospital, Bonheiden, Belgium; 5 Department of Development and Regeneration, University of Leuven, Louvain, Belgium; 6 Department of Obstetrics and Gynecology, Catharina Hospital, Michelangelolaan 2, 5623 EJ Eindhoven, the Netherlands; 7 Department Obstetrics and Gynecology, Ghent University Hospital, Oost-Vlaanderen, Corneel Heymanslaan 10, 9000 Ghent, Belgium; 8 Department of Human Structure and Repair, Ghent University, Ghent, Belgium

**Keywords:** Box training, Laparoscopy, Single-port surgery, Surgical skills, vNOTES

## Abstract

**Objective:**

With the growing adoption and implementation of vaginal Natural Orifice Transluminal Endoscopic Surgery (vNOTES), structured hands-on simulation practice is essential for the training of gynecologists and residents. This study compares the performance of validated surgical tasks using a conventional multiport laparoscopy box and a single-port vNOTES box. Additionally, participants’ experiences and task load for each training modality were evaluated.

**Design, setting and participants:**

This multi-center, prospective comparative study included three groups: medical students and inexperienced gynecological residents (*n =* 15), gynecological residents (*n =* 15), and gynecologists (*n =* 15). Participants performed four validated laparoscopic skill tests on both the conventional laparoscopy box and the vNOTES box. For each task and box, total task time, number of errors, and total scores were recorded. Questionnaires regarding baseline characteristics, preferred box for hand–eye coordination, depth perception, and instrument handling, as well as task load (NASA Task Load Index scores) for both boxes were evaluated.

**Results:**

A total of 45 participants were included, 35 females (77.8%) and 10 males (22.2%), with a mean age of 34.2 years (SD 11.1). Across all four tasks, the use of the vNOTES box was associated with less favorable scores in terms of task time, error rates, and total scores compared to the conventional laparoscopy box. In addition, the laparoscopy box was favored for depth perception (86.7%), hand–eye coordination (91.1%) and instrument usage (97.8%). The vNOTES box was associated with higher scores across all NASA Task Load Index domains, indicating it was more demanding overall.

**Conclusion:**

This study demonstrates that performing standardized surgical tasks using the vNOTES technique is significantly more challenging than with conventional laparoscopy, resulting in higher task load and inferior performance across all experience levels. These findings underline the need for tailored training, as existing laparoscopic skills do not directly translate to vNOTES proficiency. Future research should develop and validate vNOTES simulation exercises.

**Supplementary Information:**

The online version contains supplementary material available at 10.1007/s00464-025-12389-7.

With the increasing implementation of vaginal Natural Orifice Transluminal Endoscopic Surgery (vNOTES) worldwide, training methods for gynecologists and residents are essential. Single-incision laparoscopic surgery (SILS) involves technical difficulties, including reduced triangulation and instrument mobility, increased instrument collisions, and impaired depth perception, making the procedure more challenging for many surgeons [1, 2].

Studies about the learning curve for SILS showed that operating time decreases with more experience, without increasing complication rates [2, 3]. Regarding the learning curve for the vNOTES hysterectomy, surgical competence was reached after 20 cases for a well-trained gynecological endoscopist [4, 5]. Experienced gynecologists in laparoscopy will exponentially improve their surgical performance between 10 and 25 cases [5]. To ensure safe implementation and effective training, deeper insight into the learning curve and training difficulties is needed. Simulation box training can contribute to vNOTES skill development before starting the actual surgical learning curve, by providing a structured learning environment [6]. Simulation-based training surpasses traditional clinical education in skill acquisition, enabling trainees to refine both psychomotor and cognitive skills while adjusting case complexity [6–9].

Despite growing interest in vNOTES, simulation-training for this technique has not been developed and studied. Due to differences in depth perception and movement planes, vNOTES is expected to be more challenging than laparoscopy. This study compared the performance of validated surgical tasks on a vNOTES box versus a conventional laparoscopy box across three groups with different surgical experience. Additionally, we evaluated the difficulty of the exercises, the preferred box regarding depth perception, hand–eye coordination and instrument usage and the perceived task load.

## Materials and methods

### Study design and participants

This multi-center, prospective comparative study was conducted at the Zuyderland Medical Center and Catharina Hospital Eindhoven, The Netherlands. The study was approved on 23 June 2022 by the Medical Review Ethics Committee and registered as a clinical trial (METCZ20220051). Participants were divided into three groups based on laparoscopic experience: Group 1 (Novice) included 15 inexperienced gynecological residents and medical students; Group 2 (Intermediate) consisted of 15 experienced gynecological residents; and Group 3 (Advanced) included 15 practicing gynecologists. None of the participants had prior experience with vNOTES. Baseline characteristics, including sex, age, years of experience in gynecology, and laparoscopic experience, were collected for all participants.

### Outcome measures and data collection

The primary objective of this study was to compare the performance of validated surgical tasks (based on differences in scores) between the vNOTES box and the conventional laparoscopy box in three groups with varying laparoscopic experience. The secondary objective was to evaluate user experience (including task difficulty and box preference) and task load associated with performing exercises on the two boxes.

**Fig. 1:**
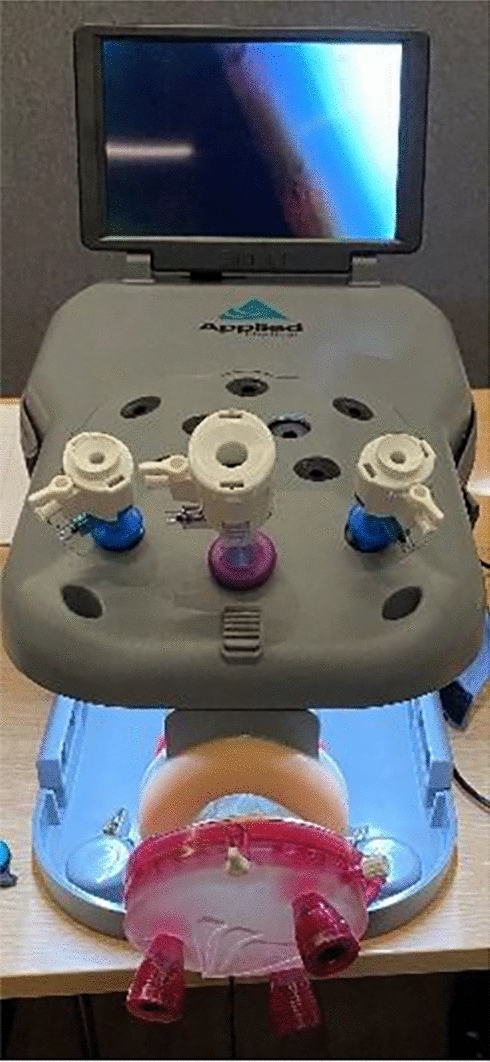
Laptrainer

**Fig. 2:**
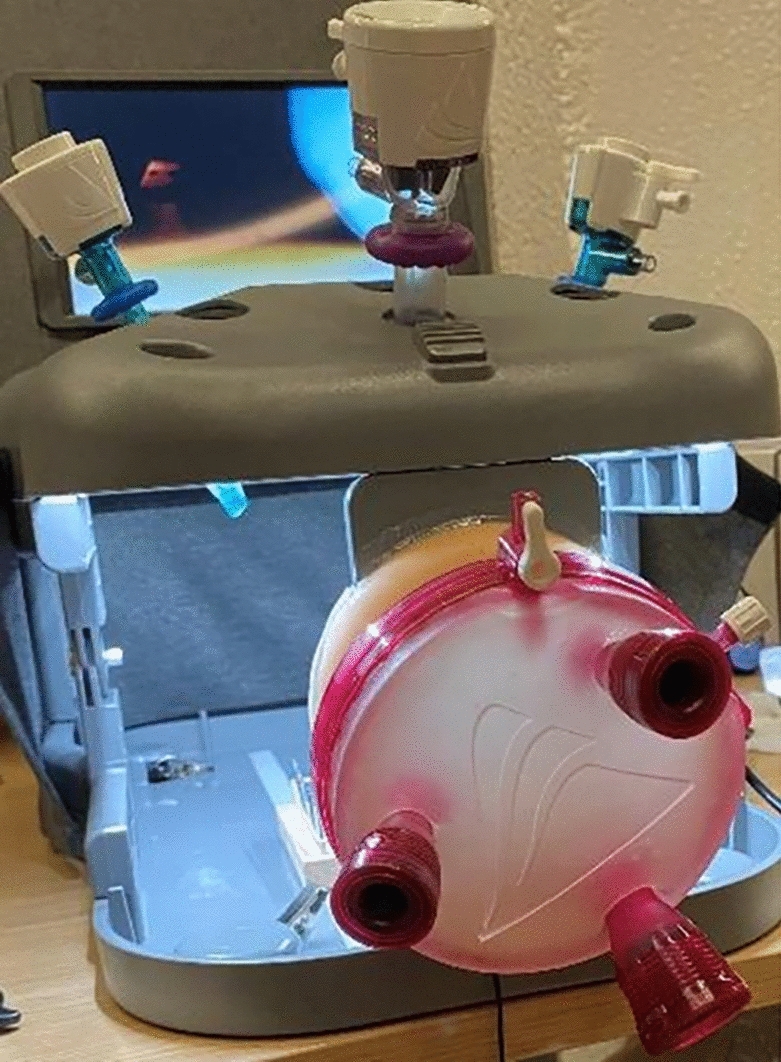
GelPOINT V-Path on Laptrainer

Each participant completed four standardized exercises in a fixed sequence during each session, with the starting modality (laparoscopy or vNOTES box) alternated between participants. Each exercise was performed once on each simulation box without prior practice. Exercises with the laparoscopy box were done standing, those with the vNOTES box seated. Surgical skills were assessed using the SIMSEI Laptrainer Non-Tissue with 5 mm × 35 cm EPIX LAPA instruments (Applied Medical, Netherlands) (Fig. 1). For vNOTES, a 9.5 cm GelPOINT V-Path Platform was used (Fig. 2). A 0° endoscopic camera was inserted through the lowest port of either the Laptrainer or the GelPOINT V-Path. In the vNOTES box, the camera was held by either the instructor or the participants, neither of whom had prior experience with vNOTES.

**Fig. 3:**
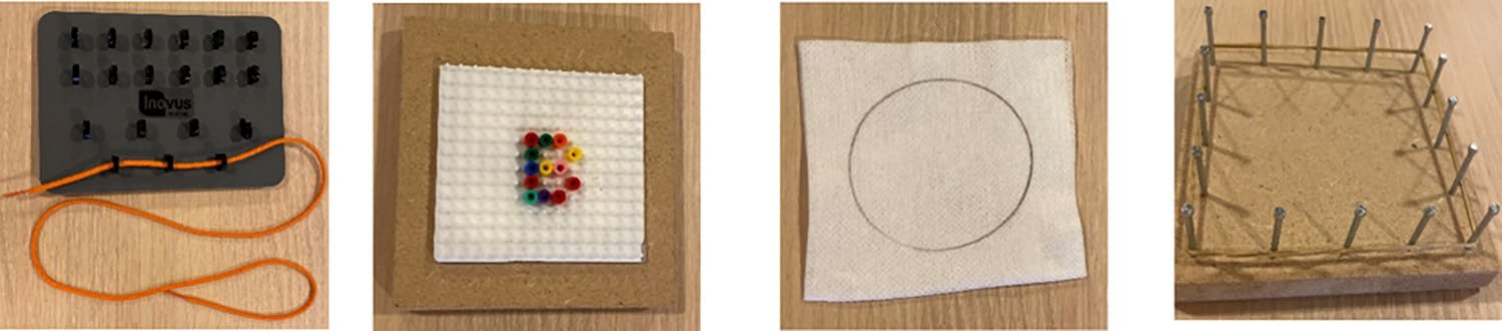
The exercises: pipe cleaner, letter B, cut the circle, and elastic band

Participants performed four validated exercises for conventional laparoscopy—pipe cleaner, letter B, cut the circle, and elastic band (Fig. 3) [10]—as detailed in Appendix A. These same tasks were used on the vNOTES box, as no standardized vNOTES-specific exercises currently exist. Each task had a 10-minute time limit and was assessed by proctors and participants based on errors and scores validated by Kolkman [10]. Task time and numbers of errors were recorded. The total scores were calculated by time in seconds + (number of errors × 10) (Appendix A). Differences in time, errors, and total scores were compared between the two training boxes and among the three groups.

After completing the exercises, all participants filled out two quantitative questionnaires comparing the single-port and conventional multiport techniques. The first questionnaire was a self-constructed questionnaire with multiple-choice questions. This questionnaire assessed exercise difficulty (easiest versus hardest exercise) and box preference. The box preference was based on hand–eye coordination, depth perception and instrument handling (laparoscopy or vNOTES).

The second questionnaire was the NASA Task Load Index (NASA-TLX), which evaluates task load across six dimensions: mental, physical, and temporal demand, performance, effort, and frustration [11]. Scores range from 0 to 100 in 5-point increments, with higher scores indicating greater task load—except for ‘performance’, where higher scores reflect lower perceived effectiveness. Raw (unweighted) scores were recorded per box for each dimension. The following questions were answered:

- Mental demand: How mentally demanding was the task?
- Physical demand: How physically demanding was the task?
- Temporal demand: How hurried or rushed was the pace of the task?
- Performance: How successful were you in accomplishing what you were asked to do?
- Effort: How hard did you have to work to accomplish your level of performance?
- Frustration: How insecure, discouraged, irritated, stressed, and annoyed were you?

### Statistical analysis

Data were analyzed using SPSS version 29 (IBM SPSS Statistics for Windows, Version 29.0.2.0 Armonk, NY: IBM Corp). Baseline characteristics and questionnaires were summarized with univariate descriptive analysis: continuous data as mean (SD), and ordinal/nominal variables as frequencies (%). Non-parametric tests were used due to non-normal data distribution. Within-group differences were analyzed by the Related-Samples Wilcoxon signed rank test. Between-group differences were calculated by an independent-samples Kruskal–Wallis test. For pairwise comparisons between the groups, Mann–Whitney U-tests were used. The significance level (alpha) was adjusted by the Bonferroni correction for multiple comparisons. A significance level α (family-wise error rate) of 0.05 was used.

## Results

### Demographic characteristics

Table 1 presents the demographic characteristics of the study population. A total of 45 participants were included, comprising 35 females (77.8%) and 10 males (22.2%), with a mean age of 34.2 years (SD 11.1). None of the participants had prior experience with vNOTES. The intermediate group had a mean of 3.9 years of laparoscopic experience, with 66.6% having performed more than 30 procedures. The advanced group, some of whom had multiple subspecialties, had a mean of 10.7 years of laparoscopic experience, with 93.3% having performed over 100 procedures (Table 1).

**Table 1:** Demographic characteristics

	Novice (*n =* 15)	Intermediate (*n =* 15)	Advanced (*n =* 15)
Sex, n (%)			
Female Male	10 (66.7%) 5 (33.3%)	15 (100%) 0 (0%)	10 (66.7%) 5 (33.3%)
Age, mean (SD)	24.7 (2.0)	30.9 (2.9)	46.9 (9.9)
Years of experience, mean (range)	–	3.9 (1–6)	10.7 (1–27)
Subspecialization (*n*)			
Urogynecology Benign/Minimally invasive Oncology Fertility	– – – –	– – – –	4 7 4 3
Number of performed laparoscopy, n (%)			
0 < 10 10–30 30–50 50–100 > 100	15 (100%) – – – – –	2 (13.3%) 1 (6.7%) 2 (13.3%) 8 (53.3%) 2 (13.3%) 0 (0%)	0 (0%) 0 (0%) 0 (0%) 1 (6.7%) 0 (0%) 14 (93.3%)

### Comparative analysis of total scores for vNOTES versus laparoscopy by group and task

Performance on each of the four exercises was evaluated by comparing total scores between the vNOTES and laparoscopy boxes within the three groups (Table 2). Lower total scores indicate better performance. A positive median difference score (median vNOTES score minus median laparoscopy score) indicates better performance by laparoscopy, while a negative median difference favors vNOTES.

**Table 2:** Total scores on validated surgical tasks per group and box

Task	Group	vNOTES box	Laparoscopy box	Difference	p-value
Pipe cleaner	Novice Intermediate Advanced	240 (186–451) 119 (95–192)^B^ 177 (119–395)	155 (106–278) 53 (36–73)^B^ 59 (41–108)^C^	105 (42–246) 62 (29–108) 111 (88–354)	.012 .003 < .001
Letter B	Novice Intermediate Advanced	630 (591–640) 437 (303–630)^B^ 504 (409–605)^A^	444 (370–590) 225 (175–240)^B^ 262 (193–338)^AC^	138 (40–198) 173 (110–375) 262 (88–307)^A^	< .001 < .001 < .001
Cut the circle	Novice Intermediate Advanced	1064 (802–1535) 721 (548–829)^B^ 735 (478–1088)	873 (665–1115) 805 (630–892) 574 (448–719)^C^	56 (-257–550) -51 (-331–91) 74 (-63–515)	.392 .211 .156
Elastic band	Novice Intermediate Advanced	82 (75–100) 63 (35–89) 68 (45–100)	57 (42–67) 38 (28–53) 41 (31–54)	30 (13–49) 27 (-14–58) 29 (6–46)	.015 .088 .031

*n =* 15 per group. Total scores and differences within the groups are displayed as median (p25-p75). Lower median scores indicate a better total score. Note that the median difference score does not always match the difference in median scores

^A ^*n =* 14 due to missing data

^B ^Significantly different: novice versus intermediate per box

^C ^Significantly different: novice versus advanced per box

Significantly higher total scores for the vNOTES box compared to the conventional laparoscopy box were observed in the *pipe cleaner* task across all three groups. Median score differences were 105 (IQR: 42–246, *p* = 0.012) in the novice group, 62 (IQR: 29–108, *p* = 0.003) in the intermediate group, and 111 (IQR: 88–354, *p* < 0.001) in the advanced group (Table 2). A similar significant pattern was observed in the *letter B* task, with median score differences of 138 (IQR: 40–198), 173 (IQR: 110–375), and 262 (IQR: 88–307) in the novice, intermediate, and advanced group, respectively (p < 0.001 for all). No significant differences were found between the two simulation boxes for the *cut the circle* task in any group. For the *elastic band* task, significantly higher total scores with the vNOTES box compared to the laparoscopy box were observed in the novice group (median difference 30 [IQR: 13–49], *p* = 0.015) and the advanced group (median difference 29 [IQR: 6–46], *p* = 0.031). In the intermediate group, the median difference (27 [IQR: 14–58], *p* = 0.088) was not statistically significant (Table 2).

Overall, these findings indicate inferior performance on the vNOTES box compared to the conventional laparoscopy box for the pipe cleaner and letter B tasks across all groups, as well as for the elastic band task in both the novice and advanced groups.

#### Analysis of between-group differences per task and box

Additional analyses were conducted to compare the performance between groups within each training box. Notably, no significant differences in total scores were observed between the intermediate and advanced groups on either box, although the intermediate group generally showed slightly better scores. Significant between-group differences were observed, particularly between the novice and intermediate group for both boxes, as well as between the novice and advanced group for the laparoscopy box. In all cases, the novice group demonstrated lower performance compared to the other groups (Table 2). For detailed time scores and errors, see Appendix B.

#### Exercise difficulty and box preference

Table 3 summarizes exercise difficulty ratings per box. In both boxes, the elastic band was rated as the easiest exercise (vNOTES: 77.8%, laparoscopy: 88.9%), while the Letter B was the most challenging (vNOTES: 82.2, laparoscopy: 62.2%) (Table 3).

**Table 3:** Exercise difficulty ratings by box

Exercise	Easiest vNOTES (%)	Hardest vNOTES (%)	Easiest laparoscopy (%)	Hardest laparoscopy (%)
Pipe cleaner	6.7% (*n =* 3)	15.6% (*n =* 7)	11.1% (*n =* 5)	8.9% (*n =* 4)
Letter B	2.2% (*n =* 1)	82.2% (*n =* 37)	–	62.2% (*n =* 28)
Cut the circle	13.3% (*n =* 6)	2.2% (*n =* 1)	–	28.9% (*n =* 13)
Elastic band	77.8% (*n =* 35)	–	88.9% (*n =* 40)	–

The percentages and corresponding sample sizes (n) indicate the proportion of participants who rated each exercise as the easiest or the hardest

Furthermore, the laparoscopy box was preferred for depth perception (*n =* 39; 86.7%), hand–eye coordination (*n =* 41; 91.1%), and instrument usage (*n =* 44; 97.8%).

### Analysis of NASA task load index scores (NASA-TLX)

Across all three groups, vNOTES was associated with significantly higher scores on all NASA-TLX domains compared to conventional laparoscopy, except for the ‘performance’ item, where no significant difference was observed (Table 4).

**Table 4:** Scores on all six domains of NASA Task Load Index scale per group and box

Domain NASA-TLX	Group	vNOTES box	Laparoscopy box	Difference	p-value
Mental demand	Novice Intermediate Advanced	80 (75–85) 70 (65–90) 70 (50–80)	65 (40–75) 50 (25–70) 50 (20–60)	20 (10–30) 25 (10–40) 20 (0–35)	< .001 .001 .005
Physical demand	Novice Intermediate Advanced	60 (40–80) 75 (55–85) 65 (45–75)	40 (25–45) 40 (20–60) 50 (20–65)	25 (0–30) 35 (0–55) 20 (0–35)	.003 .010 .008
Temporal demand	Novice Intermediate Advanced	80 (60–85) 65 (35–85) 55 (30–70)	45 (35–65) 50 (25–65) 50 (25–60)	25 (0–30) 10 (0–40) 10 (0–15)	.003 .018 .140
Performance	Novice Intermediate Advanced	55 (50–70) 70 (45–75) 60 (35–75)	55 (30–75) 45 (30–75) 30 (25–65)	20 (-15–30) 0 (-15–45) 5 (-5–25)	.277 .207 .091
Effort	Novice Intermediate Advanced	80 (75–90) 80 (75–90) 75 (60–90)	60 (60–75) 60 (40–70) 55 (45–65)	15 (5–25) 20 (10–40) 15 (10–35)	.002 .002 < .001
Frustration	Novice Intermediate Advanced	75 (65–85) 80 (60–90) 65 (50–75)	40 (25–60) 40 (25–60) 30 (15–40)	25 (10–35) 25 (15–45) 35 (15–45)	< .001 .001 .001

*n =* 15 per group. All data are displayed as median (p25-p75). Scores range from 0 (low) – 100 (high), except performance is 0 (high) – 100 (low). Note that the median difference score does not always match the difference in median scores

## Discussion

This study demonstrates that the overall performance of standardized surgical tasks using the vNOTES technique is more challenging and inferior compared to the conventional multiport laparoscopy. These differences were observed even among novices with no prior surgical experience. The majority of participants preferred the laparoscopy box due to better depth perception, hand–eye coordination, and instrument handling. All three groups scored higher across all NASA-TLX domains when using vNOTES, indicating a higher task load. It is well established that structured training and repeated exposure to laparoscopic box trainers lead to improved surgical skills and increased confidence [12, 13]. Interestingly, our findings show that residents outperformed gynecologists in most exercises, likely due to the integration of box training as a standard component of their gynecological residency program.

This study highlights that, despite existing laparoscopic skills, vNOTES requires distinct technical and surgical competencies. The single-port technique limits triangulation, thereby increasing procedural difficulty [1, 2]. These findings are consistent with previous studies comparing performances in SILS versus conventional laparoscopy. Marcus et al. found that novice, intermediate, and expert surgeons performed significantly worse in single-port surgery compared to multiport surgery (*p *< 0.1) when completing basis laparoscopic tasks such as peg transfer and pattern cutting [14]. Similarly, Cox et al. reported that medical students in the single-port group took longer and required more repetitions to achieve proficiency in these tasks than those in the multiport group [15]. Two other studies focused on skill retention in novice surgeons performing laparoscopic tasks on a SILS box and a (multiport) laparoscopy box [1, 16]. They found that SILS skills were retained for four weeks but declined after eight and twelve weeks. In contrast, laparoscopic skills were maintained throughout the twelve-week period, highlighting the need for continuous training in SILS [1].

This study is the first multi-center, quantitative, prospective comparative study, comparing surgical skills using a vNOTES box and a conventional laparoscopy box among participants with varying levels of surgical experience. It provides further insight into the task load associated with both training boxes.

However, this study has several limitations. The exercises used were validated for conventional laparoscopy, not for vNOTES, which undermines the construct validity of both the tasks and the vNOTES box. Exercises that are not tailored to vNOTES-specific features may inadequately assess relevant skills. Moreover, if the box fails to realistically simulate vNOTES procedures, it compromises the overall validity of the simulation, potentially affecting the accuracy of training and study outcomes. The vNOTES scores may also have been influenced by inexperienced camera operators. Interobserver bias was present due to the absence of trained or standardized assessors. Although this bias was consistent across all groups, it still represents a methodological limitation. Furthermore, the sample size was relatively small (15 participants per group), which limits generalizability, although it is comparable to other box training studies. vNOTES experts were not included in this study, since vNOTES was only recently introduced in the Netherlands. Lastly, in this study, only the domain ‘performance’ did not reach statistical significance. This is likely due to participants misunderstanding of the reverse scoring system for this item (with 0 indicating optimal performance and 100 indicating poor performance), in contrast to the other NASA-TLX domains.

The lack of standardized vNOTES exercises highlights a critical gap in surgical training. Single-port surgery is technically more challenging, requires more time to achieve proficiency, and is more difficult to master and retain. Simulation and box training are essential for acquiring and mastering new surgical techniques. Therefore, the development of standardized exercises specifically for vNOTES is necessary to optimize training outcomes. It is also important to support gynecologists and residents in learning this surgical technique. Currently, the integration of vNOTES into gynecological residency programs is not standardized. Although residents may have opportunities to perform vNOTES procedures during their training, no effective or validated training routines are currently available. Moreover, it should be noted that standardized training exercises designed for conventional laparoscopy may not effectively translate to vNOTES proficiency.

Effective vNOTES exercises should focus on hand–eye coordination due to confined operating spaces and limited triangulation. It must also improve depth perception and spatial orientation, especially for movements across horizontal and vertical planes. This contrasts with multiport laparoscopy, where a superior perspective reduces triangulation challenges. Further research is needed to identify, design, and validate vNOTES-specific training exercises that address the unique challenges posed by this technique.

## Conclusion

This study demonstrates that performing standardized surgical tasks using the vNOTES technique is significantly more challenging than with conventional multiport laparoscopy, resulting in higher task load and inferior performance across all experience levels. These findings underline the need for tailored training, as existing laparoscopic skills do not directly translate to vNOTES proficiency. The lack of validated vNOTES-specific exercises and standardized integration into residency programs presents a critical gap in current surgical education. To address the unique demands of single-port surgery, future research should focus on developing and validating simulation-based training that emphasizes spatial orientation, depth perception, and hand–eye coordination.

## Supplementary Information

Below is the link to the electronic supplementary material.

Supplementary file1 (DOCX 247 KB)

Supplementary file2 (DOCX 202 KB)
