# Supplementary material for: Surgical skills assessment on a vaginal Natural Orifice Transluminal Endoscopic Surgery (vNOTES) simulation box compared to a conventional endoscopic simulation box; the SAVE trial
Source: Surg Endosc. 2026 Mar 2;40(5):3936–43. doi: 10.1007/s00464-025-12389-7 (PMC13161292; doi:10.1007/s00464-025-12389-7)
Supplement: Supplementary file 1 — Supplementary file1 (DOCX 247 KB) [file 464_2025_12389_MOESM1_ESM.docx]

**Supplementary appendix A**

*Standardized laparoscopic exercises*

**Pipe cleaner**

**
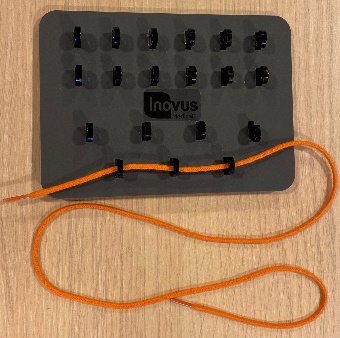
**The goal was to get the rope through all the three hooks. The exercise starts when the rope was picked up with the grasper. The exercise was completed when the rope passed all three hooks (see picture). Errors were given with every missed hook. Two graspers were used. The total score was calculated: by time in seconds + (number of errors x 10).

**Letter B**


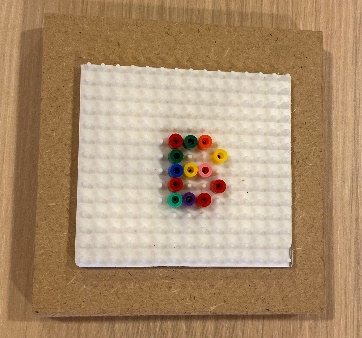
The goal of this exercise was to create a letter B with the beads. The beads were picked up with the dominant hand using graspers; the non-dominant hand could help while creating the letter B. The exercise started when the first bead was picked up and finished when the letter B was complete. Every dropped bead next to the floor and outside of the vision field was scored as one error. Two graspers were used. The total score was calculated: by time in seconds + (number of dropped beads x 10).

**Cut the circle**

**
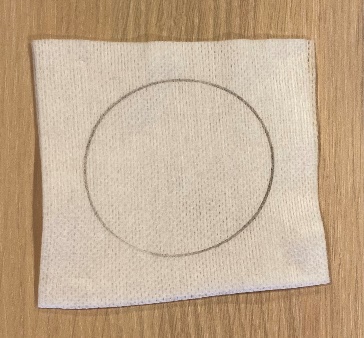
**In this exercise the circle drawn on the gauze had to be cut out. The exercise started with the first cut and ended when the circle was released from the gauze. The surface away from the drawn line (in millimeters) was registered as an error. The participants were allowed to use one grasper and one scissor. The total score was calculated: by time in seconds + (the cut surface in mm away from the drawn line x 10).

**Elastic band**

**
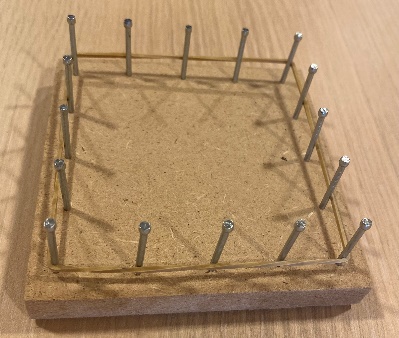
**The goal of this exercise was to stretch the rubber band around all nails. The exercise started from the moment the rubber band was grabbed and ended when the rubber band was stretched around all nails. Every missed nail was scored as an error. Here, two graspers were used. The total score was calculated: by time in seconds + (number of missed nails x 10).
