# Supplementary material for: Surgical skills assessment on a vaginal Natural Orifice Transluminal Endoscopic Surgery (vNOTES) simulation box compared to a conventional endoscopic simulation box; the SAVE trial
Source: Surg Endosc. 2026 Mar 2;40(5):3936–43. doi: 10.1007/s00464-025-12389-7 (PMC13161292; doi:10.1007/s00464-025-12389-7)
Supplement: Supplementary file 2 — Supplementary file2 (DOCX 202 KB) [file 464_2025_12389_MOESM2_ESM.docx]

**Supplementary appendix B**

*Boxplots per task, box, and group: total scores, time scores, and number of errors.* *All scores are displayed as median (p25-p75). Lower total scores indicate better scores. Additional table with p-values for between group differences.*


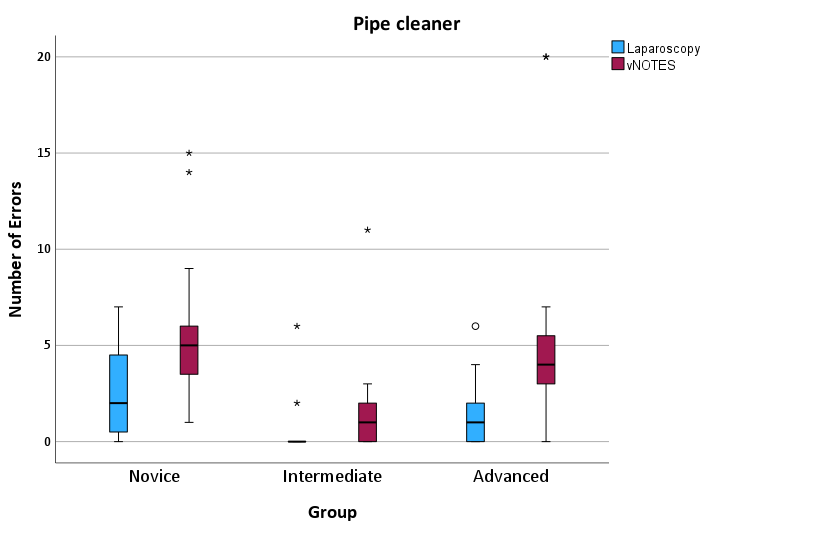

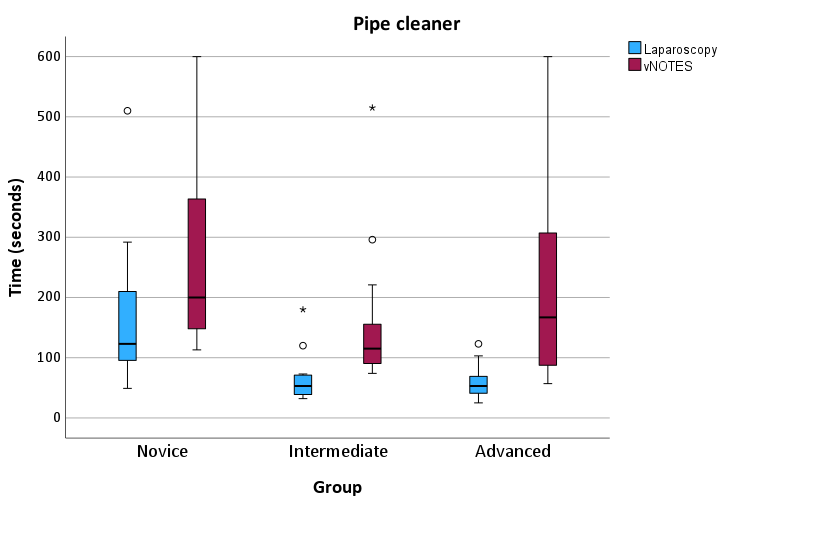

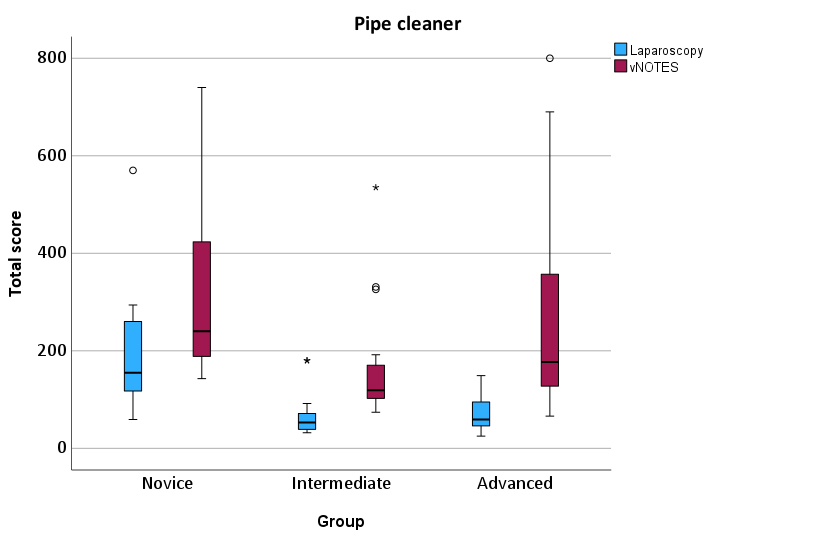
**Figure 1. Pipe cleaner: total scores, time scores, and number of errors**


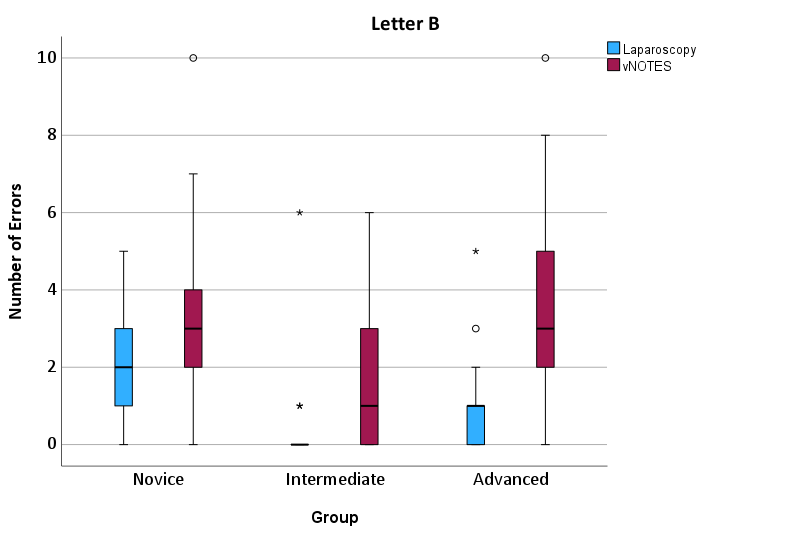

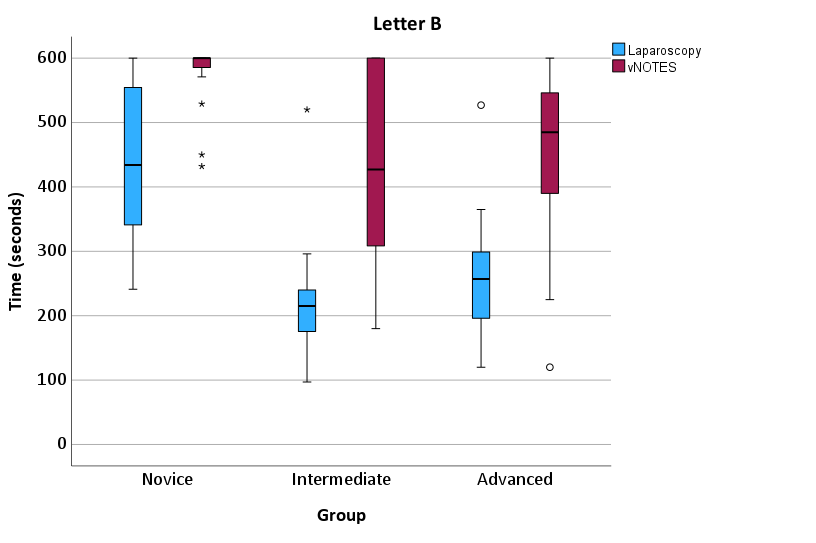

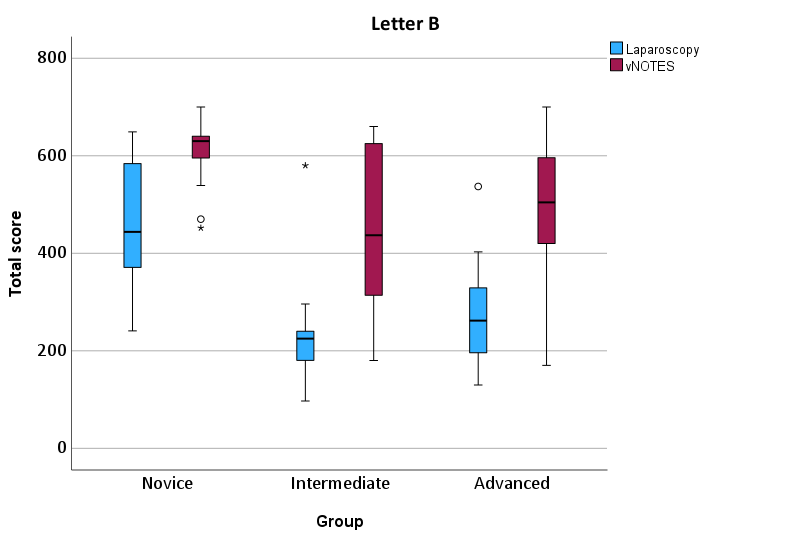
**Figure 2. Letter B: total scores, time scores, and number of errors**


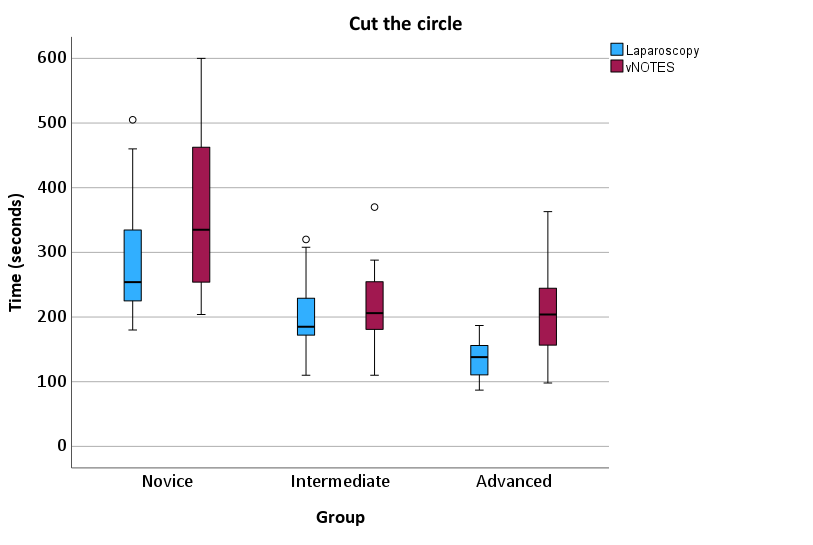

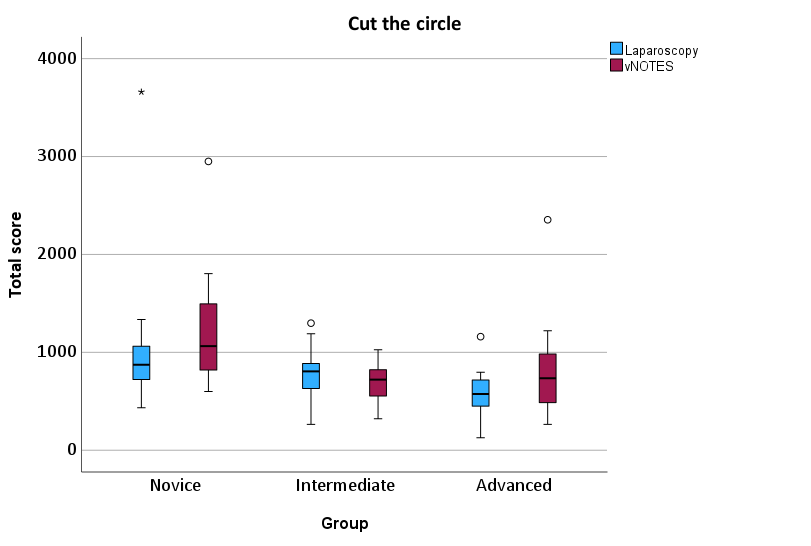
**Figure 3. Cut the circle: total scores, time scores, and number of errors**


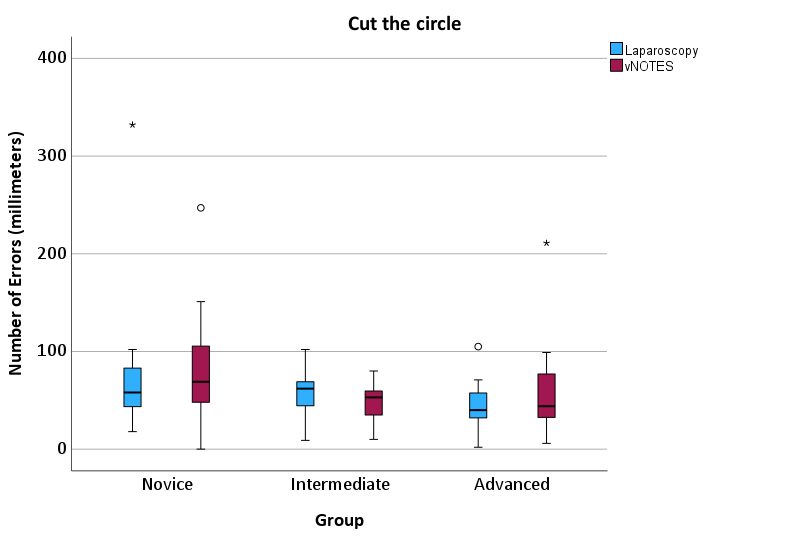


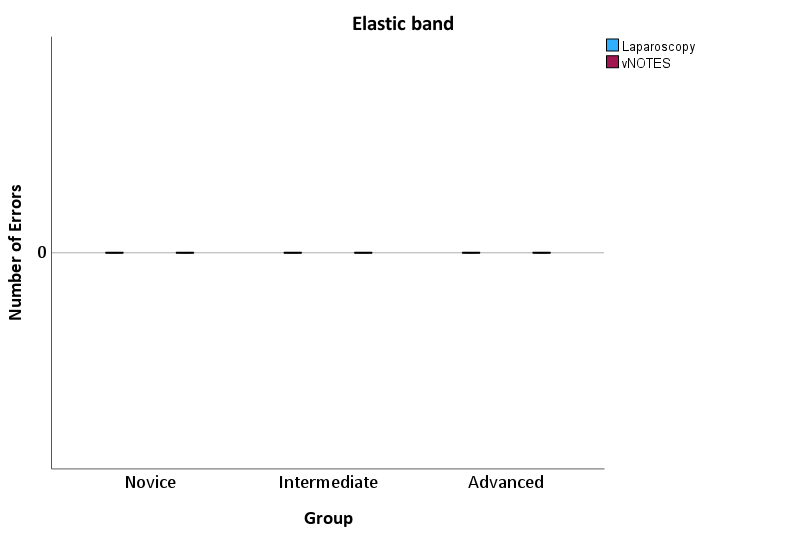

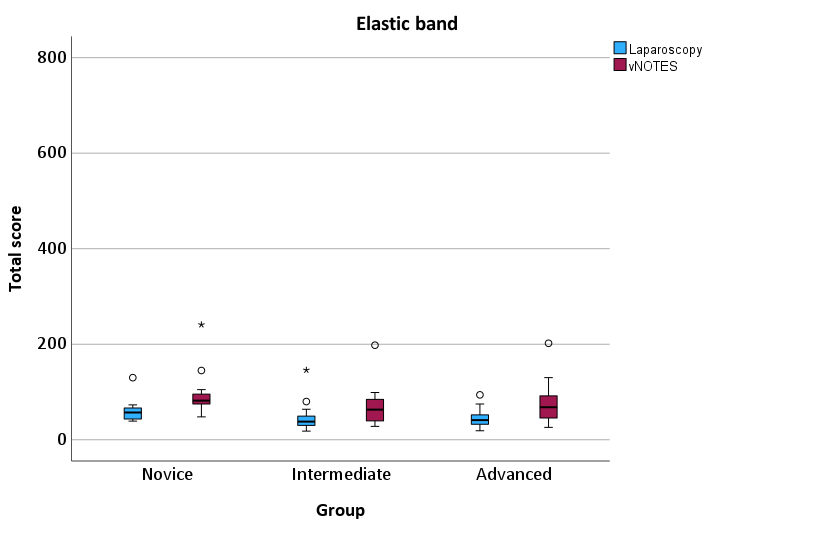
**Figure 4. Elastic band: total scores = time scores, and number of errors**

*No errors were made in this task; therefore, total scores and time scores are identical.*

**Table 1. Between-group differences in total scores per task and box.**

| Task | Box | Novice (1) | Intermediate (2) | Advanced (3) | Group  1 vs 2 | | Group  1 vs 3 | | Group  2 vs 3 |  |
| --- | --- | --- | --- | --- | --- | --- | --- | --- | --- | --- |
| Pipe cleaner | vNOTES  Laparoscopy | 240 (186-451)  155 (106-278) | 119 (95-192)  53 (36-73) | 177 (119-395)  59 (41-108) | p=.008  p=.001 | | p=.416  p=.002 | | p=.384  p=1.000 |  |
| Letter B | vNOTES  Laparoscopy | 630 (591-640)  444 (370-590) | 437 (303-630)  225 (175-240) | 504 (409-605)*  262 (193-338)* | p=.043  p=<.00 | | p=.090  p=.002 | | p=1.000  p=.992 |  |
| Cut the circle | vNOTES  Laparoscopy | 1064 (802-1535)  873 (665-1115) | 721 (548-829)  805 (630-892) | 735 (478-1088)  574 (448-719) | p=.012  p=.853 | | p=.060  p=.005 | | p=1.000  p=.115 |  |
| Elastic band | vNOTES  Laparoscopy | 82 (75-100)  57 (42-67) | 63 (35-89)  38 (28-53) | 68 (45-100)  41 (31-54) | p=.145  p=.05 | p=.307  p=.152 | | p=1.000  p=1.000 | | |
